# CircNEIL3 promotes cervical cancer cell proliferation by adsorbing miR-137 and upregulating KLF12

**DOI:** 10.1186/s12935-020-01736-4

**Published:** 2021-01-07

**Authors:** Yuan Chen, Yiting Geng, Junchao Huang, Dan Xi, Guoping Xu, Wendong Gu, Yingjie Shao

**Affiliations:** 1 Department of Radiation Oncology, The Third Affiliated Hospital of Soochow University, 185 Juqian Street, Changzhou, 213003 China; 2 Department of Oncology, The Third Affiliated Hospital of Soochow University, Changzhou, 213003 China

**Keywords:** circNEIL3, miR-137, KLF12, Cervical cancer

## Abstract

**Background:**

CircRNAs play crucial roles in multiple tumours. However, the functions of most circRNAs in cervical cancer remain unclear.

**Methods:**

This study collected GSE113696 data from the GEO database to search for differentially expressed circRNAs in cervical cancer. Quantitative reverse transcription PCR was used to detect the expression level of circNEIL3 in cervical cancer cells and tissues. Then, functional experiments in vitro and in vivo were performed to evaluate the effects of circNEIL3 in cervical cancer.

**Results:**

CircNEIL3 was highly expressed in cervical cancer. In vivo and in vitro experiments verified that circNEIL3 enhanced the proliferation capacity of cervical cancer cells. RNA immunoprecipitation, luciferase reporter assay, pull-down assay, and fluorescent in situ hybridization confirmed the interaction between circNEIL3 and miR-137 in cervical cancer. A luciferase reporter assay showed that circNEIL3 adsorbed miR-137 and upregulated KLF12 to regulate the proliferation of cervical cancer cells.

**Conclusions:**

CircNEIL3 is an oncogene in cervical cancer and might serve as a ceRNA that competitively binds to miR-137, thereby indirectly upregulating the expression of KLF12 and promoting the proliferation of cervical cancer cells.

## Background

Cervical cancer is the second most common malignant tumour in females worldwide and has severely threatened the physical health of females [1]. Cervical cancer is also an important cause of cancer-related death among women worldwide [2]. There are approximately 529,000 new cervical cancer cases annually, resulting in 260,000 deaths [2]. Human papillomavirus (HPV) vaccines have greatly contributed to the primary prevention of cervical cancer, but in some developing countries, the prevention of cervical cancer can hardly be realized due to the cost and limited availability [3, 4]. At present, the clinical therapeutic strategies for cervical cancer include surgical resection, radiotherapy, chemotherapy, or combined therapy. Although substantial progress has been attained in the diagnosis and treatment of cervical cancer, the prognosis for cervical cancer patients is still dismal, with a 5-year overall survival (OS) rate of lower than 30% in most countries [5]. Therefore, it is important to investigate the initiation and development mechanisms of cervical cancer, to develop novel diagnostic and prognostic biomarkers, and to find therapeutic targets for tumour prevention.

In 1976, Sanger first discovered circular RNA (circRNA) in viroids [6]. With the emergence of new generation sequencing technology and the advancement of bioinformatics, the regulatory roles of circRNAs in eukaryotic cells have gradually been recognized [7, 8]. CircRNA is a kind of noncoding RNA (ncRNA) with a ring structure made through covalent binding that is not affected by RNA excision enzymes and is evolutionally conserved. Its multiple biological functions, such as chromatic remodelling, transcription and post-transcription processing, and gene expression regulatory functions have been extensively investigated and reported, which mainly manifest at three levels: the epigenetic level (chromosome silencing, genomic imprinting and chromatin modification), the transcription level and the post-transcription level. CircRNA has become a hot topic in genetic research [9–11]. It has been indicated that circRNAs play crucial roles in the initiation and development of multiple tumours, including cervical cancer [12–17]. However, the functions of most circRNAs in cervical cancer remain unclear. This study collected GSE113696 data from the GEO database to search for differentially expressed circRNAs in cervical cancer [12]. Data from cervical cancer patient specimens from the Third Affiliated Hospital of Soochow University were combined with GEO data, and it was discovered that circNEIL3 was highly expressed in cervical cancer. In vivo and in vitro experiments verified that circNEIL3 enhanced the proliferation capacity of cervical cancer cells. Subsequent molecular mechanism analysis revealed that circNEIL3 was an oncogene that served as the ceRNA to indirectly upregulate KLF12 expression by competitively binding with miR-137, thus promoting the proliferation of cervical cancer cells.

## Methods

### Cells and tissues

A normal cervical epithelial cell line (End1/E6E7) and cervical cancer cell lines (SiHa, C-4 I, HeLa and C-33A) were purchased from American Type Culture Collection (ATCC, Manassas, VA, USA). All cells were cultured in RPMI 1640 containing 10% foetal bovine serum (FBS) and incubated in the cell incubator at 37 °C and 5% CO_2_.

Postoperative specimens were collected from 20 cervical cancer patients receiving surgical resection at the Third Affiliated Hospital of Soochow University from 2016 to 2017. After surgery, the specimens were pathologically confirmed as cervical cancer tissues and matched with para-carcinoma normal tissues. All cervical cancer patients received radiochemotherapy or chemotherapy. The specimens were immersed in liquid nitrogen immediately after surgery and then transferred into an 80 °C refrigerator for preservation. This study protocol conformed to the Declaration of Helsinki. All tests were approved by the Ethics Committee of the Third Affiliated Hospital of Soochow University. The patients and family members were informed of the pathology results of the specimens and signed written informed consent forms.

### Cell transfection

The circNEIL3 overexpression plasmid, circNEIL3 small interfering RNA (siRNA) and negative control plasmids were purchased from Generbiol (Shanghai, China). Moreover, miR-137 mimics, miR-137 inhibitors and negative controls were obtained from RiboBio (Guangzhou, China). Lipofectamine 3000 was utilized for transfection in accordance with the instructions.

### Quantitative real-time reverse transcription PCR (qRT-PCR)

TRIzol reagent was used to extract tissue and cellular total RNA, and the total RNA content and concentration were determined by using a NanoDrop 2000 microspectrophotometer. Then, the PrimeScript RT Reagent Kit was utilized to reverse transcribe the total RNA into cDNA, and TB Green Fast qPCR Mix was used for qRT-PCR. GAPDH was used as the internal reference for circRNA and mRNA, while U6 was used as the internal reference for miRNA. The comparative Ct (2^−ΔΔCt^) method was used to calculate the expression quantities of circRNA, mRNA and miRNA. The information for all primers is presented in Additional file 1: Table S1.

### Western blotting

RIPA was used to lyse cells, and total cellular protein was extracted. Thereafter, the BCA method was adopted to determine the cellular protein concentration, and an equivalent amount of sample was collected from each group for sodium dodecyl sulfate-polyacrylamide gel electrophoresis (SDS-PAGE), and the proteins were transferred onto membranes. Later, the membranes were blocked for 1 h, and then the KLF12 or GAPDH antibody was added and incubated at 4 °C overnight. Then, the membranes were washed with TBST, incubated with secondary antibody for 1 h, washed, and developed with ECL, followed by X-ray film exposure, development, fixation, and scanning. Finally, the results were calculated.

### CCK-8

A total of 100 µl of transfected cell suspension (approximately 1 × 10^3^ cells) was inoculated into 96-well plates and cultured. At the monitoring time points (24, 48, 72 and 96 h), 10 µl of CCK-8 solution was added into each well, and the culture plate was gently tapped for sufficient mixing to avoid the production of bubbles that affect reading OD values. After incubation at 37 °C for 2 h, the OD value was measured with Bio-Tek EPOCH2 at 450 nm. The test was repeated three times.

### Colony formation assay

The transfected cells were prepared into cell suspensions, inoculated into culture dishes supplemented with 10 ml of preheated (37 °C) culture medium, and cultured in a 37 °C incubator containing 5% CO_2_ for 14 days. Then, the cells were washed with PBS twice, fixed with 4% paraformaldehyde for 20 min, and treated with 0.1% crystal violet for 30 min at room temperature. The number of cell colonies with a diameter of > 0.1 mm was observed under the microscope.

### Actinomycin D transcription inhibition assay

The SiHa cells were divided into 5 aliquots and placed into 5 wells (wells 1–5) of 24-well plates. At 24 h, cells in well 1 were harvested, and RNA was extracted. Actinomycin D (Abcam, abl41058) was added to wells 2–5 at a final concentration of 2 µg/ml. After 4, 8, 12 and 24 h, cells in wells 2–5 were collected to extract the RNA for subsequent qRT-PCR.

### Luciferase reporter assays

The wild-type and mutant sequences were synthesized and cloned into dual-luciferase reporter gene plasmids (Promega) containing the psiCheck2 promoter to biologically predict the possible miRNA target genes and binding sequences. After SiHa cells were inoculated into a 96-well plate and cultured for 24 h, the cells were co-transfected with wild-type or mutant reporter gene plasmids and overexpression or silencing plasmid mimics. Luciferase activity was measured 48 h after transfection.

### RNA immunoprecipitation (RIP)

RNA enrichment was examined by qRT-PCR using a Magna RIP RNA binding protein immunoprecipitation kit (Millipore, Billerica, MA, USA) according to the manufacturer’s instructions using anti-Ago2 or a control IgG as the antibody.

### Fluorescence in situ hybridization (FISH)

SiHa cells were used for FISH to determine the subcellular localization of circNEIL3 and miR-137. The cell specimens were pre-denatured in PBS containing 10% stationary liquid for 5 min. Then, the slide specimens were immersed into the stationary liquid twice for 10 min, processed with ice-cold ethanol at gradient volume fractions of 70%, 90% and 100%, and dried. Later, 50 ml of 50% formamide/2 × SSC was added into the wet box and preheated at 37 °C. Finally, the FITC-labelled probe circNEIL3 and the PE-labelled probe miR-137 (Tingzhou; Shanghai, China) were detected and observed by confocal microscopy. The probe sequences are displayed in Additional file 1: Table S2.

### Subcutaneous tumour formation in nude mice

Altogether, 10 4-week-old female BALB/c nude mice were randomly divided into the si-circNEIL3 group and si-NC group, with 5 mice in each group. Later, 1 ml (approximately 5 × 10^7^ cells) of SiHa cell suspension transfected with si-circNEIL3 or si-NC was inoculated subcutaneously into one side of the axilla in mice in the si-circNEIL3 group and in the si-NC group. The general conditions of nude mice were observed and recorded, and the xenograft formation time and size were observed at 3-day intervals. After the completion of the experiment, the tumours were completely dissected, photos of the entire tumours were taken, and tumour volume and weight were measured. All animal experiments were performed in line with the Animal Care and Laboratory Guidelines and approved by the Ethics Committee of the Third Affiliated Hospital of Soochow University.

### Statistical analysis

SPSS 22.0 statistical software was adopted for statistical analysis. GraphPad Prism 5.0 and Adobe Photoshop CS5 software were employed for producing images. Experimental data are expressed as the mean ± standard error of the mean (mean ± SEM). Differences between two groups were compared by Student’s t-test, while those among multiple groups were analysed by one-way analysis of variance (ANOVA). A difference of P < 0.05 indicated statistical significance; otherwise, there was no significant difference. Each experiment was repeated at least three times.

## Results

### Different expression of circRNAs in cervical cancer

**Fig. 1:**
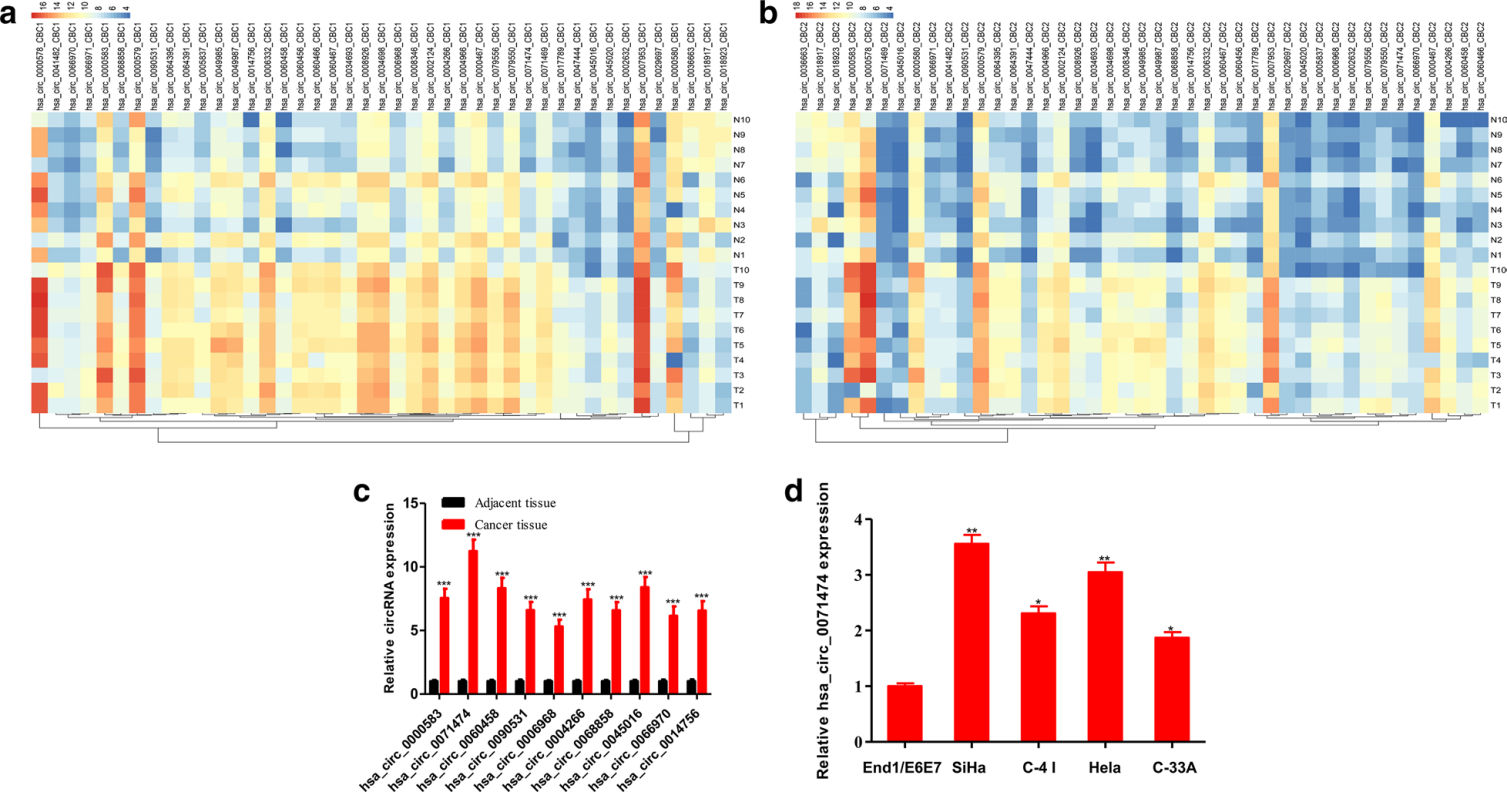
Screening of differentially expressed circRNAs in cervical cancer. **a** Analysis of differentially expressed circRNAs in cervical cancer based on GSE90737 dataset detected by the long probe (30 nt) (Fold change > 2.0, *P* < 0.05 in t-test). **b** Analysis of differentially expressed circRNAs in cervical cancer based on GSE90737 dataset detected by the short probe (20 nt) (Fold change > 2.0, *P* < 0.05 in t-test). **c** Validation of circRNAs in 20 pairs of cervical cancer tissues and para-carcinoma tissues via qRT-PCR. **d** The expression of hsa_circ_0071474 in the cervical cancer cell lines SiHa, C-4 I, C-33A, HeLa and the normal cervical epithelial cells End1/E6E7 was detected by qRT-PCR. *P < 0.05, **P < 0.01, ***P < 0.001

To investigate the differentially expressed circRNAs between cervical cancer and para-carcinoma tissues, the GSE113696 dataset was used [12]. The GSE90737 dataset provided the high-throughput ChIP data of circRNA in 10 pairs of cervical cancer and para-carcinoma tissues (CapitalBio Technology Human CircRNA Microarray v2.0). The circRNA microarray used two probes with different lengths (long probe: 30 nt; short probe: 20 nt). The long probe (30 nt) has a high detection signal, while the short probe (20 nt) has high specificity. In our study, using the criteria of fold change > 2.0 and t-test *P* < 0.05, the long probe detected the abnormal expression of 4125 circRNAs, while the short probe detected the abnormal expression of 4229 circRNAs. Among them, 1613 circRNAs were abnormally expressed by both the long probe and the short probe. Subsequently, the differential circRNAs were screened from these 1613 circRNAs, among which 43 circRNAs with a fold change > 4.0 were detected by both the long probe and the short probe (Fig. 1a, b), including 40 upregulated and 3 downregulated circRNAs. The top 10 circRNAs with the most significant differential expression in the 20 pairs of cervical cancer and para-carcinoma tissues (hsa_circ_0000583, hsa_circ_0071474, hsa_circ_0060458, hsa_circ_0090531, hsa_circ_0006968, hsa_circ_0004266, hsa_circ_0068858, hsa_circ_0045016, hsa_circ_0066970, and hsa_circ_0014756) were selected. Upon qRT-PCR verification, we discovered that hsa_circ_0071474 was the most significantly upregulated circRNA in cervical cancer tissues, and the expression increased by 11.37-fold (Fig. 1c). Moreover, we detected hsa_circ_0071474 expression in a normal cervical epithelial cell line (End1/E6E7) and cervical cancer cell lines (SiHa, C-4 I, HeLa and C-33A). The results suggested that hsa_circ_0071474 was highly expressed in cervical cancer cell lines (Fig. 1d).

### Features of circNEIL3

**Fig. 2:**
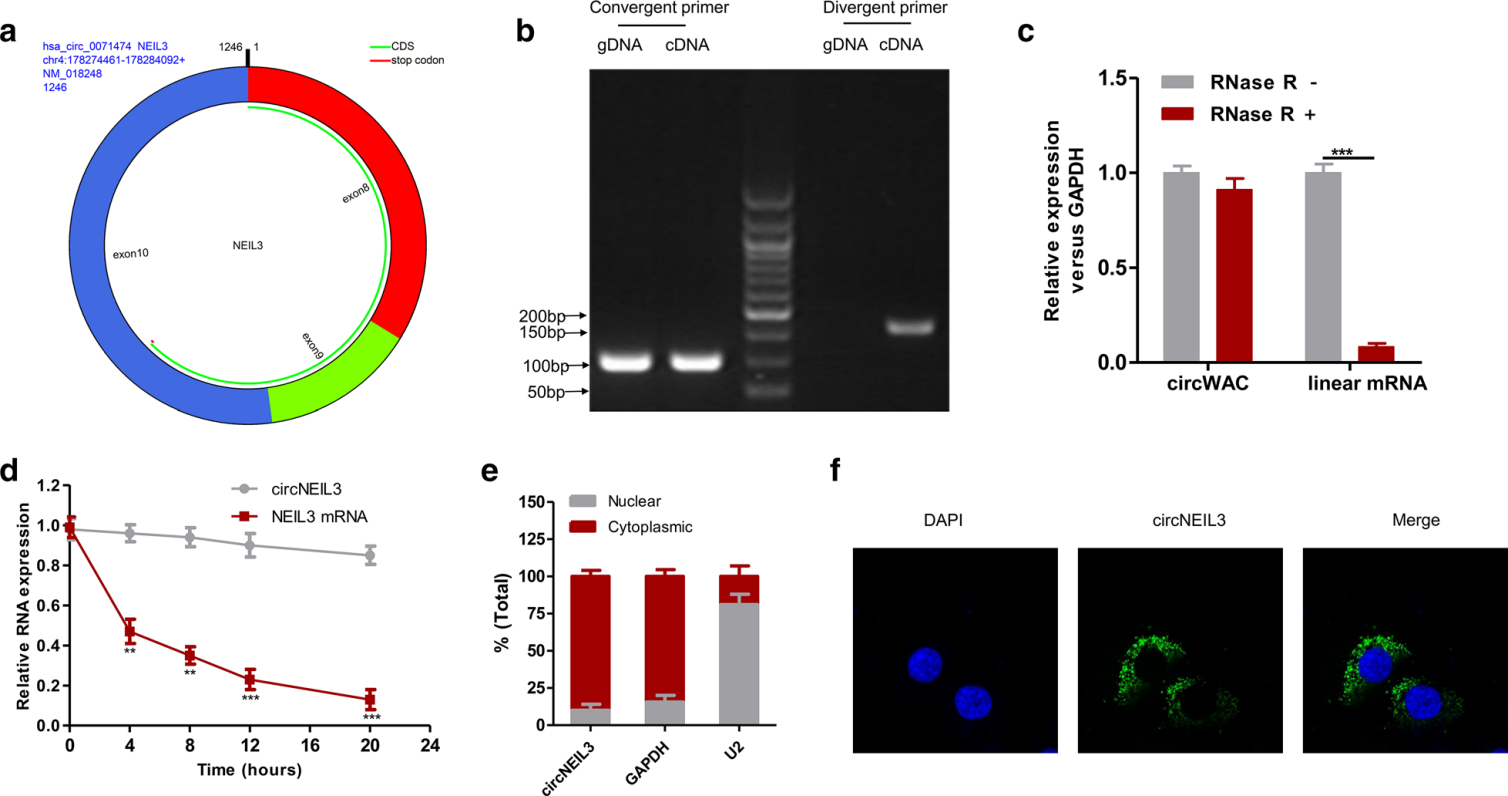
The features of circNEIL3 in cervical cancer. **a** The exonic information of circNEIL3 was illustrated as indicated. **b** qRT-PCR assay with convergent or divergent primers indicating the existence of circNEIL3 in SiHa cells. **c** The expressions of circNEIL3 and linear mRNA in SiHa cells were detected by qRT-PCR in the presence or absence of RNase R. **d** qRT–PCR analysis for the expression of circNEIL3 and NEIL3 mRNAs after treatment with Actinomycin D in SiHa cells. **e** Nuclear-cytoplasmic fractionation experiment showing that circNEIL3 was mainly distributed in the cytoplasm. **f** FISH for circNEIL3. Nuclei were stained with DAPI. Data were represented as means ± S.D. of at least three independent experiments. *P < 0.05, **P < 0.01, ***P < 0.001

Using the circBase database (http://www.circbase.org/), it was suggested that hsa_circ_0071474 was formed by the cyclization of exons 8–10 in the NEIL3 gene, with a spliced length of 1246 bp (hereafter referred to as circNEIL3) (Fig. 2a). CircRNA is a kind of closed annular base sequence formed by reverse shear pairing; therefore, in addition to PCR amplification by forward primers, reverse primers at the interface can also be used for PCR amplification. cDNA obtained through the reverse transcription of circNEIL3 and the directly extracted genomic gDNA were subjected to PCR amplification using normally designed forward primers, while only the former might be subjected to PCR amplification using reverse primers, and there was no obvious band of gDNA. This result suggested that the fragment was indeed derived from circNEIL3 (Fig. 2b). Linear RNA has poor resistance to RNase R, and the abundance of linear RNA after RNase R digestion remarkably decreased. In contrast, the annular RNA had high resistance to RNase R. After RNase R digestion, the results indicated that circNEIL3 expression was not significantly downregulated, while the linear RNA level markedly declined (Fig. 2c). Subsequently, an actinomycin D experiment was conducted, and the results suggested that the half-life of circNEIL3 was over 24 h, while that of the corresponding linear transcript was approximately 4 h (Fig. 2d). The above experiment verified that the circNEIL3 sequence was indeed circRNA. In addition, by nuclear-cytoplasmic fractionation and FISH, it was determined that circNEIL3 was mainly located in the cytoplasm of SiHa cells (Fig. 2e, f).

### circNEIL3 enhanced cervical cancer cell growth

**Fig. 3:**
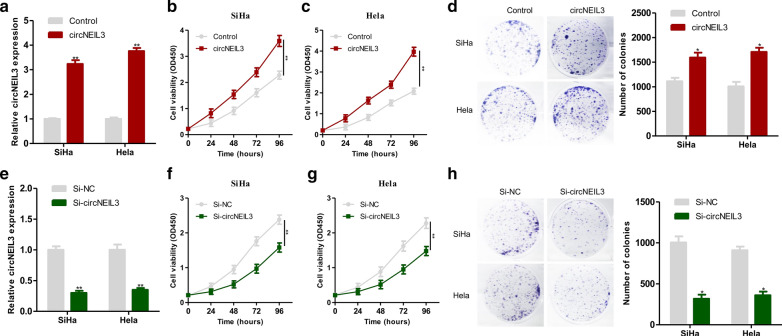
CircNEIL3 could enhanced cervical cancer cell proliferation. **a** The efficiency of circNEIL3 overexpression in SiHa and Hela cells was verified by qRT-PCR. **b**, **c** circNEIL3 enhanced cervical cancer cell growth CCK-8 proliferation assays of SiHa and Hela cells transfected with circNEIL3 or control vector. **d** Colony-formation assays of SiHa and Hela cells transfected with circNEIL3 or control vector. **e** The efficiency of circNEIL3 down expression in SiHa and Hela cells was verified by qRT-PCR. **f**, **g** CCK-8 proliferation assays of SiHa and Hela cells transfected with si-circNEIL3 or si-NC. (H) Colony-formation assays of SiHa and Hela cells transfected with with si-circNEIL3 or si-NC. Data were represented as means ± S.D. of at least three independent experiments. *P < 0.05, **P < 0.01, ***P < 0.001

Previously, we detected the expression of circNEIL3 in cervical cancer cell lines, among which the expression of circNEIL3 in SiHa and HeLa cell lines was the most significantly upregulated. Therefore, the SiHa and HeLa cell lines were selected for subsequent functional experiments. Briefly, the circNEIL3 overexpression plasmid was transfected into the SiHa and HeLa cell lines, and qRT-PCR was carried out to verify the overexpression efficiency (Fig. 3a). CCK-8 and colony formation assays revealed that the upregulation of circNEIL3 expression significantly enhanced the proliferation capacities of SiHa and HeLa cells (Fig. 3b, d). Then, circNEIL3 siRNA was transfected into the SiHa and HeLa cell lines, and qRT-PCR was conducted to verify the interference efficiency (Fig. 3e). CCK-8 and colony formation assays suggested that the downregulation of circNEIL3 expression led to significantly decreased proliferation of SiHa and HeLa cell lines (Fig. 3f, h).

### circNEIL3 adsorbed miR-137

**Fig. 4:**
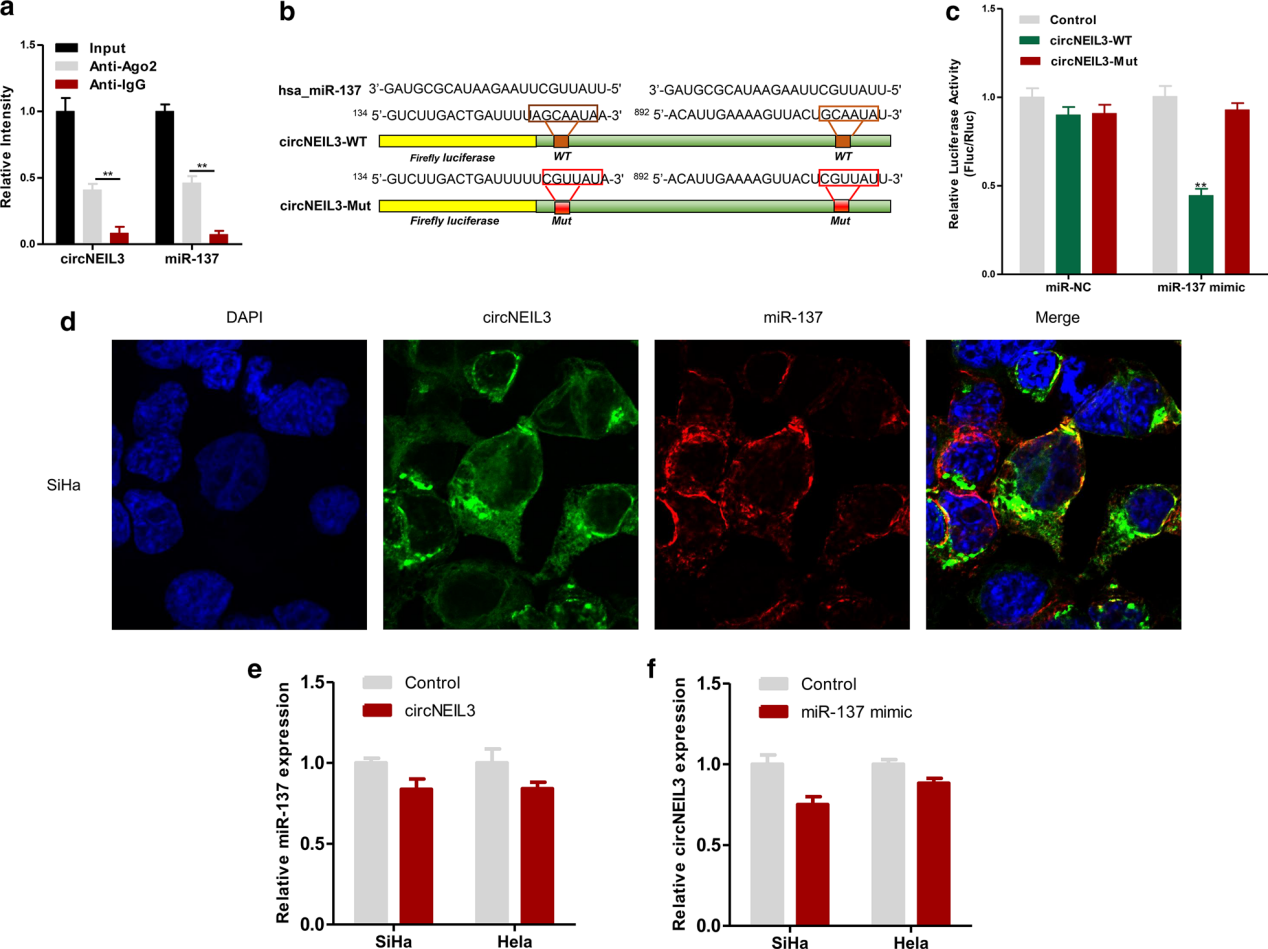
CircNEIL3 serves as a sponge for miR-137 in cervical cancer. **a** RIP experiments were performed in SiHa cells, and the co-precipitated miR-137 was subjected to qRT-PCR for circNEIL3. **b** MiR-137 and circNEIL3 binding sequences and circNEIL3 mutation sequences. **c** Luciferase activity in SiHa cells co-transfected with luciferase reporter containing wild-type or mutant circNEIL3 sequences and miR-137 mimic or control. **d** FISH showed that circNEIL3 and miR-137 were co-localized in the cytoplasm of SiHa cells. **e** After upregulation of circNEIL3 expression in the SiHa and Hela cell lines, the miR-137 expression was detected by qRT-PCR. **f** After upregulation of miR-137 expression in the SiHa and Hela cell lines, the circNEIL3 expression was detected by qRT-PCR. Data were represented as means ± S.D. of at least three independent experiments. *P < 0.05, **P < 0.01, ***P < 0.001

Previous research revealed that circNEIL3 is a kind of RNA that stably exists in the cytoplasm (Fig. 2e, f). Previous literature reports that circRNA in the cytoplasm can adsorb miRNAs [9]. Therefore, this study explored whether circNEIL3 bound with miRNAs to regulate the proliferation of cervical cancer cells. The circBank (http://www.circbank.cn/index.html) was utilized to predict the possible target genes of circNEIL3, and it was discovered that 104 miRNAs might bind with circNEIL3. Then, the most likely binding miRNAs, namely, miR-1197, miR-18a-3p, miR-1243, miR-137, miR-19a-5p, and miR-33a-5p, were screened. Later, the above miRNAs were subjected to RIP detection in SiHa cells. As a result, compared with the levels in circNEIL3 and miR-137 in the IgG antibody control group, higher levels of circNEIL3 and miR-137 were enriched in the Ago2 antibody group (Fig. 4a). This result demonstrates that circNEIL3 binds to miR-137 in the SiHa cervical cancer cell line. To further show this result, a dual-luciferase reporter gene assay was conducted. The constructed full-length circNEIL3-WT and circNEIL3-MUT luciferase vectors were co-transfected with miR-137 into SiHa cells (Fig. 4b). MiR-137 overexpression significantly reduced the activity of luciferase containing the circNEIL3-WT vector but did not reduce that of luciferase containing empty vector or circNEIL3-MUT vector (Fig. 4c). This finding verified that circNEIL3 directly bound with miR-137, and the binding site was the mutation site. To observe the colocalization of circNEIL3 and miR-137 in cells, FISH assays were carried out. The results indicated that circNEIL3 and miR-137 were colocalized in SiHa cells, mainly in the cytoplasm (Fig. 4d). In addition, when circNEIL3 was overexpressed in cervical cancer cell lines, the miR-137 expression quantity was not markedly changed (Fig. 4e); similarly, when miR-137 was overexpressed in cervical cancer cell lines, the expression quantity of circNEIL3 showed no obvious changes (Fig. 4f). These results indicated that the binding of circNEIL3 with miR-137 did not affect the expression quantity of each other. Therefore, circNEIL3 adsorbed miR-137.

### circNEIL3 adsorbed miR-137 and upregulated KLF12 to regulate the proliferation of cervical cancer cells

**Fig. 5:**
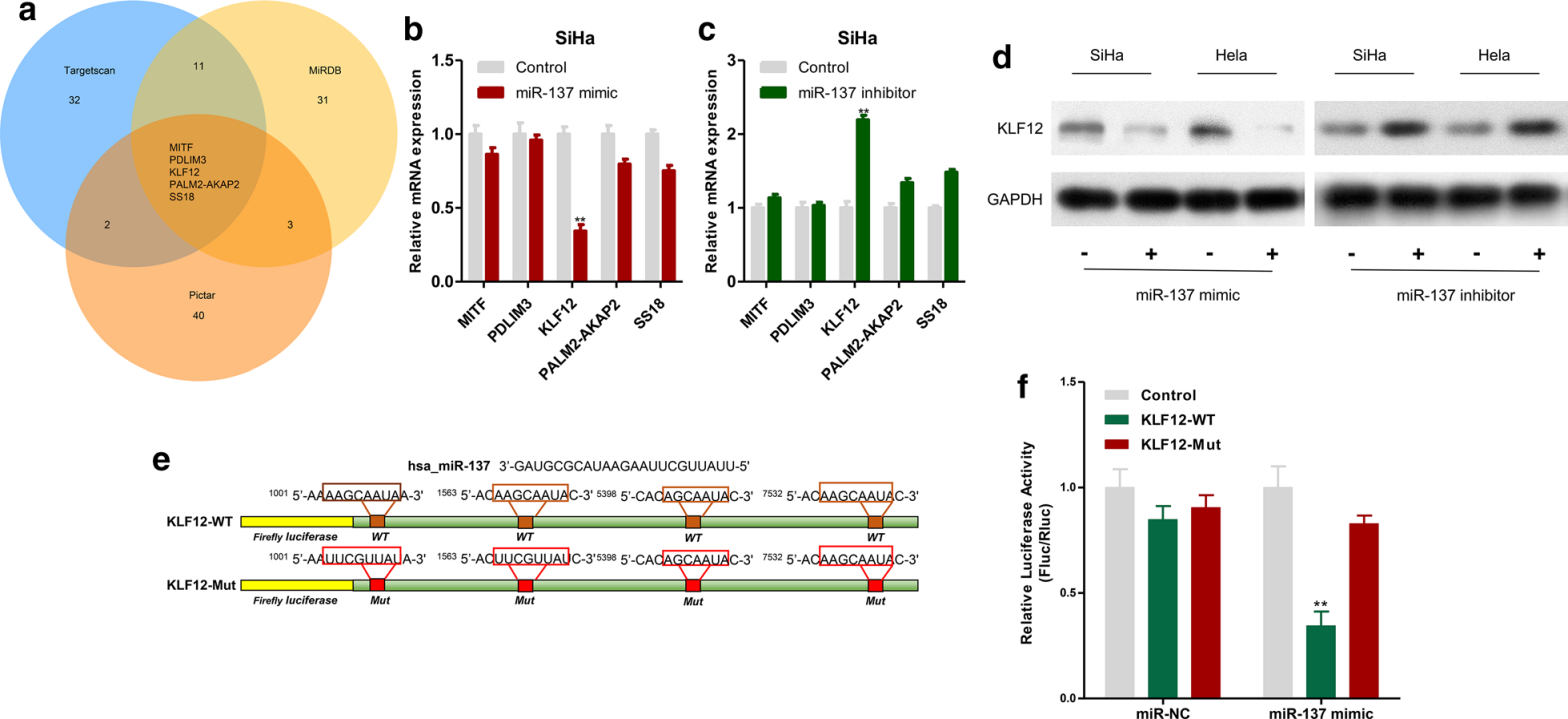
KLF12 was one of the direct target gene of miR-137. **a** TargetScan, miRDB and Pictar were used to predict the potential target genes of miR-137. **b** After upregulation of miR-137 expression in SiHa cell lines, the candidate mRNA expression was detected by qRT-PCR. **c** After downregulation of miR-137 expression in SiHa cell lines, the candidate mRNA expression was detected by qRT-PCR. **d** The expressions of KLF12 protein in SiHa and Hela transfected with miR-137 mimic or miR-137 inhibitor were measured by Western blotting. **e** MiR-137 and KLF12 binding sequences and KLF12 mutation sequences. **f** Luciferase activity in SiHa cells co-transfected with luciferase reporter containing wild-type or mutant KLF12 sequences and miR-137 mimic or control. Data were represented as means ± S.D. of at least three independent experiments. *P < 0.05, **P < 0.01, ***P < 0.001

A previous study indicated that miR-137 plays a role as a tumour suppressor gene in multiple tumours. Therefore, we speculated that circNEIL3 adsorbed miR-137 to upregulate oncogene expression in cervical cancer, thus promoting the proliferation of cervical cancer cells. The possible regulatory target genes of miR-137 were predicted by a bioinformatics approach. The top 50 predicted target genes in the TargetScan (http://www.targetscan.org/vert_72/), miRDB (http://www.mirdb.org/) and PicTar (https://pictar.mdc-berlin.de/) databases were screened and intersected to obtain the 5 shared target genes among the three databases (MITF, PDLIM3, KLF12, PALM2-AKAP2 and SS18, Fig. 5a). Afterwards, miR-137 expression was upregulated or downregulated in SiHa cells, and qRT-PCR results revealed that the mRNA expression of KLF12 was significantly changed (Fig. 5b, c). Later, KLF12 protein expression in SiHa and HeLa cells was detected by Western blotting. As a result, miR-137 overexpression downregulated the KLF12 protein expression level, while downregulating miR-137 expression elevated the KLF12 protein expression (Fig. 5d). Afterwards, we constructed the wild-type sequence (KLF12-WT) and the mutant binding sequence (KLF12-MUT) (Fig. 5e), which were co-transfected with miR-137 mimic or miR-NC into MDA-MB-231 cells for a dual-luciferase reporter assay. The results indicated that miR-137 overexpression remarkably reduced the activity of luciferase containing the KLF12-WT vector but did not reduce that of luciferase containing the KLF12-MUT vector (Fig. 5f), verifying that KLF12 was one of the direct target genes of miR-137.

**Fig. 6:**
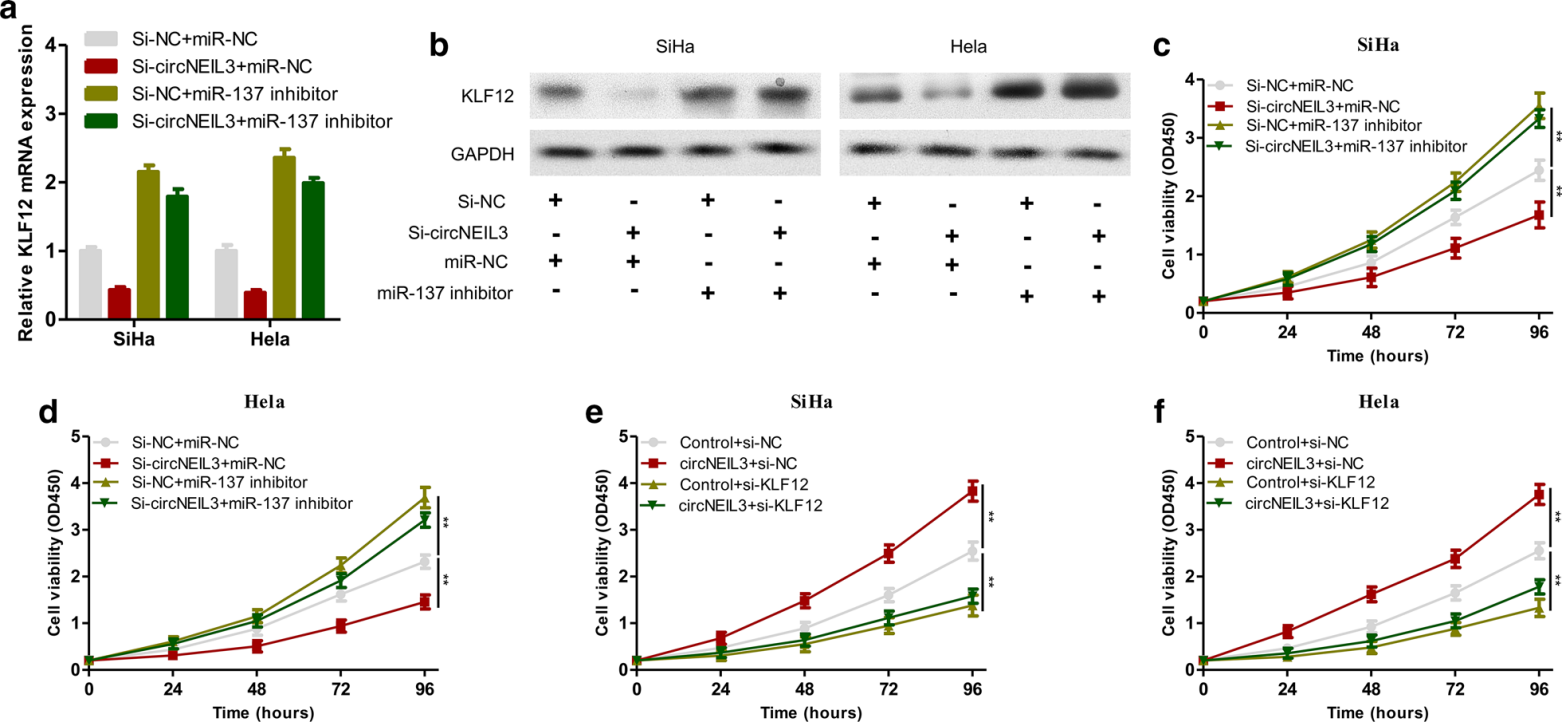
CircNEIL3 adsorbed miR-137 and up-regulated KLF12 to regulate the proliferation of cervical cancer cells. **a** The expressions of KLF12 mRNA in SiHa and Hela transfected with si-NC + miR-NC, si-circNEIL3 + miR-NC, si-NC + miR-137 inhibitor, and si-circNEIL3 + miR-137 inhibitor were detected by qRT-PCR. **b** The expressions of KLF12 protein in SiHa and Hela transfected with si-NC + miR-NC, si-circNEIL3 + miR-NC, si-NC + miR-137 inhibitor, si-circNEIL3 + miR-137 inhibitor were detected by Western blotting. **c**, **d** CCK-8 proliferation assays of SiHa and Hela cells transfected with si-NC + miR-NC, si-circNEIL3 + miR-NC, si-NC + miR-137 inhibitor, and si-circNEIL3 + miR-137 inhibitor. **e**, **f** CCK-8 proliferation assays of SiHa and Hela cells transfected with control + si-NC, circNEIL3 + si-NC, control + si-KLF12, and circNEIL3 + si-KLF12. Data were represented as means ± S.D. of at least three independent experiments. *P < 0.05, **P < 0.01, ***P < 0.001

To verify whether circNEIL3 adsorbed miR-137 and upregulated KLF12 to promote tumour proliferation, a rescue experiment was performed. In brief, SiHa and HeLa cells were subjected to the following 4 groups of transfection: (1) si-NC + miR-NC, (2) si-circNEIL3 + miR-NC, (3) si-NC + miR-137 inhibitor, and (4) si-circNEIL3 + miR-137 mimic. First, qRT-PCR and Western blotting revealed that downregulation of circNEIL3 expression suppressed KLF12 expression, while inhibiting miR-137 expression upregulated circNEIL3 expression. This result verified that circNEIL3 adsorbed miR-137 and regulated KLF12 mRNA and protein expression (Fig. 6a, b). Subsequently, the CCK-8 proliferation assay proved that, compared with the control group, circNEIL3 expression was downregulated in SiHa and HeLa cells, which suppressed cell proliferation. By contrast, in SiHa and HeLa cells, downregulating miR-137 promoted cell proliferation. However, in SiHa and HeLa cells with downregulated miR-137, the downregulation of circNEIL3 did not suppress the proliferation of these two cell lines (Fig. 6c, d). Consequently, it was proven that the role of circNEIL3 as a tumour suppressor gene in cervical cancer cells was dependent on miR-137. To determine whether circNEIL3 exerted a tumour suppression effect via KLF12, the following rescue experiment was carried out. The SiHa and HeLa cells were subjected to the following 4 groups of transfection: (1) Control + si-NC, (2) circNEIL3 + si-NC, (3) Control + si-KLF12, and (4) circNEIL3 + si-KLF12. CCK-8 cell proliferation assays revealed that, compared with the control group, overexpressing circNEIL3 in SiHa and HeLa cells promoted cell proliferation. In contrast, silencing KLF12 expression in SiHa and HeLa cells suppressed cell proliferation. However, in SiHa and HeLa cells with silenced KLF12, upregulating circNEIL3 did not promote cell proliferation (Fig. 6e, f). Therefore, the above results suggested that circNEIL3 promoted the proliferation of cervical cancer cells via KLF12. In summary, circNEIL3 is an oncogene and might serve as ceRNA, which competitively binds to miR-137, thereby indirectly upregulating KLF12 expression and promoting the proliferation of cervical cancer cells.

### Downregulating circNEIL3 suppressed the growth of cervical cancer in vivo

**Fig. 7:**
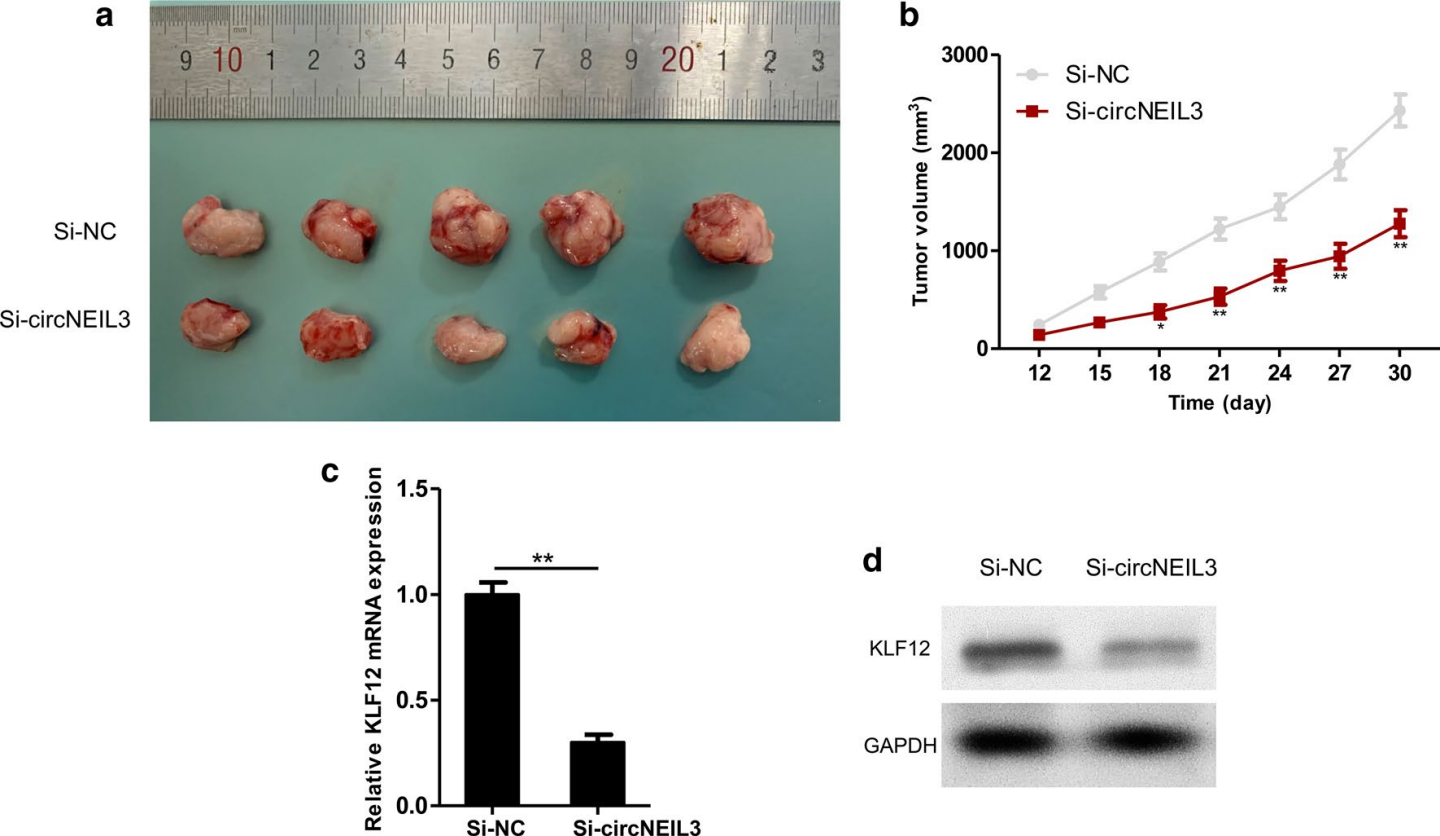
Down-regulating circNEIL3 suppressed the growth of cervical cancer in vivo. **a** Image of representative resected tumors from xenograft nude mice on the 30th day. **b** Growth curves of si-NC and si-circNEIL3. **c** The expression of KLF12 mRNA in tumor tissues detected by qRT-PCR. **d** The expression of KLF12 protein in tumor tissues detected by Western blotting. *P < 0.05, **P < 0.01, ***P < 0.001

To examine the important biological functions of circNEIL3 in the initiation and development of cervical cancer, cervical cancer tumour-bearing mice were constructed according to the indicated steps. Then, SiHa cells stably transfected with circNEIL3 siRNA or si-NC were injected subcutaneously into the backs of nude mice. Ten days after injection, the growth of subcutaneous xenografts in nude mice was observed every 3 days, the long diameter and short diameter of the tumour body were measured using a Vernier calliper, and the tumour volume was calculated. After 30 days of culture, the tumour tissues were dissected, as presented in Fig. 7a. From day 18 of injection, the subcutaneous xenograft volume in nude mice of the si-circNEIL3 group began to be significantly smaller than that in the control group (Fig. 7b). Apart from subcutaneous xenograft volume, we also detected the expression levels of KLF12 in two groups with subcutaneous xenografts. The results suggested that the KLF12 mRNA and protein expression levels in the circNEIL3 siRNA group apparently declined (Fig. 7c, d).

## Discussion

circRNA possesses high stability, conservatism and abundance, exerts multiple functions, participates in numerous biological processes [18], and may exert an important role in cancer [19]. circRNA is a promising tumour diagnostic biomarker and a novel target for antitumour treatment [19–21]. An increasing number of studies have discovered that circRNAs play a vital role in the initiation and development of cervical cancer [12–17, 22, 23]. Hu et al. discovered that circ_0067934 was overexpressed in cervical cancer tissues and cell lines, and the high expression of circ_0067934 was related to the poor prognosis of cervical cancer. circ_0067934 knockdown suppressed the proliferation and migration of cervical cancer cells. hsa_circ_0000263 adsorbed miR-150-5p to regulate the initiation and development of cervical cancer cells [23]. Cao et al. found that circPOLA2 was highly expressed in cervical squamous cell carcinoma (CSCC), which was positively correlated with the poor prognosis of CSCC patients. circPOLA2 knockdown suppressed the proliferation, migration and invasion of cervical cancer cells both in vivo and in vitro. circPOLA2 adsorbed endogenous miR-326 and promoted GNB1 expression [16]. Yang et al. discovered that circOSBPL10 was significantly upregulated in cervical cancer cells. Downregulating circOSBPL10 inhibited the proliferation and migration of cervical cancer cells. circOSBPL10 absorbed miR-1179, upregulated UBE2Q1 expression, and thus affected cervical cancer cell functions [15]. Ji et al. reported that circSLC26A4 was upregulated in cervical cancer cells and tissues. In the clinic, the high expression of circSLC26A4 is related to the low survival rate of cervical cancer patients. circSLC26A4 knockdown inhibited tumour growth, proliferation and invasion both in vitro and in vivo. Moreover, circSLC26A4 promoted the progression of cervical cancer via the miR-1287-5p/HOXA7 axis [14]. Ou et al. verified that circ-AKT1 was highly expressed in cervical cancer tissues and cells. The high expression of circ-AKT1 and AKT1 accelerated cervical cancer cell proliferation and invasion. In addition, TGF-β induced the expression of circ-AKT1 and AKT1 and promoted EMT in cervical cancer [13]. This study screened the most significant circRNA (circNEIL3) through the differential expression between cervical cancer tissues and para-carcinoma tissues. circNEIL3 was highly expressed in cervical cancer tissues and cells. It was verified that circNEIL3 was indeed the annular structure mainly distributed in the cytoplasm. Meanwhile, upregulating circNEIL3 promoted the proliferation of cervical cancer cells, whereas downregulating circNEIL3 suppressed cervical cancer cell proliferation. Animal experiments also proved that knocking out circNEIL3 suppressed tumour growth.

Subsequently, this study explored and verified the molecular mechanism of circNEIL3 in promoting cervical cancer proliferation. First, the candidate miRNAs of circNEIL3 were screened through a bioinformatics approach. RIP and dual-luciferase reporter assays confirmed that circNEIL3 adsorbed miR-137. MiR-137 is located on chromosome 1p22, which is a miRNA abundant in brain tissue, has multiple potential target genes and participates in numerous biological processes through different target genes [24]. miR-137 is downregulated in high-grade glioma, and its expression level in high-grade glioma is significantly lower than that in low-grade glioma, revealing that miR-137 is closely related to the malignant transformation of glioma. In addition, COX2 in glioma can serve as the target gene of miR-137 [25]. In addition, COX2 knockdown or miR-137 upregulation promotes the proliferation of glioma cells. Thus, it is proposed that miR-137 suppresses COX2 gene expression as a suppressor of glioma cell production [26]. Chen et al. discovered that miR-137 expression in normal tissue decreased relative to that in gastric cancer tissues, and this decline was negatively correlated with Cdc42 expression. Further research discovered that increased miR-137 expression and inactivation of Cdc42 promoted tumour cell apoptosis or cell cycle arrest at G1 phase, thus suppressing the development of gastric cancer [27]. Some research indicates that miR-137 inhibits Cdc42 and Cdk6 expression to suppress the development of non-small cell lung cancer (NSCLC) [28]. In cervical cancer tissues and cells, miR-137 expression is downregulated. The lower expression of miR-137 is related to the short OS of cervical cancer patients. Upregulating miR-137 expression suppresses cervical cancer cell proliferation and migration. EZH2 is the direct downstream target gene of miR-137 in cervical cancer [29]. Miao et al. also discovered that miR-137 was expressed at low levels in cervical cancer tissues and cells. Overexpression of miR-137 suppressed EMT in cervical cancer cells, cell proliferation, colony formation, invasion, migration and tumour formation. Further mechanistic studies verified that miR-137 bound with GREM1 to suppress the activation of the TGF-β/Smad pathway [30]. In summary, miR-137 mainly plays a role as a tumour suppressor gene in tumours. In our study, downregulating miR-137 suppressed the proliferation of SiHa and HeLa cells and reversed the decreased proliferation of cervical cancer induced by circNEIL3 downregulation. Finally, by bioinformatics approaches and cell experiments, the target gene regulated by miR-137 was identified, which proved that KLF12 expression was regulated by circNEIL3 and miR-137. In addition, rescue experiments confirmed that KLF12 blocked the effect of circNEIL3 on improving the proliferation of cervical cancer. KLF12 has been verified as an oncogene in multiple tumours [31–34].

Although this study has confirmed the regulatory function of circNEIL3/miR-137/KLF12 in cervical cancer, there are still some questions to be resolved: (1) Does circNEIL3 regulate signal pathways other than miR-137/KLF12? (2) Whether circNEIL3 regulates the proliferation of cervical cancer through mechanisms other than ceRNA? 3. Previous studies have confirmed that miR/137 can regulate KLF12 in gastric cancer and pancreatic cancer. Is there a regulatory network of circNEIL3/miR-137/KLF12 in these two tumors? All of the above needs to be explored and verified by further research in the future.

In conclusion, circNEIL3/miR-137/KLF12 can form a ceRNA network to regulate the proliferation of cervical cancer cells. Based on this mechanism, we believe that circNEIL3/miR-137/KLF12 may be a promising and novel biomarker and therapeutic target for cervical cancer.

## Supplementary Information

**Additional file 1: Table S1.** Primers for qRT-PCR. **Table S2.** RNA probes for FISH.

## Data Availability

The circRNA expression data for cervical cancer from GSE113696.

## References

[1] Bray F, Ferlay J, Soerjomataram I, Siegel RL, Torre LA, Jemal A (2018). Global cancer statistics 2018: GLOBOCAN estimates of incidence and mortality worldwide for 36 cancers in 185 countries. CA Cancer J Clin.

[2] Small W, Bacon MA, Bajaj A, Chuang LT, Fisher BJ, Harkenrider MM, Jhingran A, Kitchener HC, Mileshkin LR, Viswanathan AN (2017). Cervical cancer: a global health crisis. Cancer.

[3] Olson B, Gribble B, Dias J, Curryer C, Vo K, Kowal P, Byles J (2016). Cervical cancer screening programs and guidelines in low- and middle-income countries. Int J Gynaecol Obstet.

[4] Chen W, Zheng R, Baade PD, Zhang S, Zeng H, Bray F, Jemal A, Yu XQ, He J (2016). Cancer statistics in China, 2015. CA Cancer J Clin.

[5] Li H, Wu X, Cheng X (2016). Advances in diagnosis and treatment of metastatic cervical cancer. J Gynecol Oncol.

[6] Sanger HL, Klotz G, Riesner D, Gross HJ, Kleinschmidt AK (1976). Viroids are single-stranded covalently closed circular RNA molecules existing as highly base-paired rod-like structures. Proc Natl Acad Sci USA.

[7] Zhang XO, Wang HB, Zhang Y, Lu X, Chen LL, Yang L (2014). Complementary sequence-mediated exon circularization. Cell.

[8] Jeck WR, Sharpless NE (2014). Detecting and characterizing circular RNAs. Nat Biotechnol.

[9] Memczak S, Jens M, Elefsinioti A, Torti F, Krueger J, Rybak A, Maier L, Mackowiak SD, Gregersen LH, Munschauer M (2013). Circular RNAs are a large class of animal RNAs with regulatory potency. Nature.

[10] Hansen TB, Jensen TI, Clausen BH, Bramsen JB, Finsen B, Damgaard CK, Kjems J (2013). Natural RNA circles function as efficient microRNA sponges. Nature.

[11] Ma S, Kong S, Wang F, Ju S (2020). CircRNAs: biogenesis, functions, and role in drug-resistant Tumours. Mol Cancer.

[12] Li S, Teng S, Xu J, Su G, Zhang Y, Zhao J, Zhang S, Wang H, Qin W, Lu ZJ (2019). Microarray is an efficient tool for circRNA profiling. Brief Bioinform.

[13] Ou R, Mo L, Tang H, Leng S, Zhu H, Zhao L, Ren Y, Xu Y (2020). circRNA-AKT1 sequesters miR-942-5p to upregulate AKT1 and promote cervical cancer progression. Mol Ther Nucleic Acids.

[14] Ji F, Du R, Chen T, Zhang M, Zhu Y, Luo X, Ding Y (2020). Circular RNA circSLC26A4 accelerates cervical cancer progression via miR-1287-5p/HOXA7 axis. Mol Ther Nucleic Acids.

[15] Yang S, Jiang Y, Ren X, Feng D, Zhang L, He D, Hong S, Jin L, Zhang F, Lu S (2020). FOXA1-induced circOSBPL10 potentiates cervical cancer cell proliferation and migration through miR-1179/UBE2Q1 axis. Cancer Cell Int.

[16] Cao Y, Li J, Jia Y, Zhang R, Shi H (2020). CircRNA circ_POLA2 promotes cervical squamous cell carcinoma progression via regulating miR-326/GNB1. Front Oncol.

[17] Cai H, Zhang P, Xu M, Yan L, Liu N, Wu X (2019). Circular RNA hsa_circ_0000263 participates in cervical cancer development by regulating target gene of miR-150-5p. J Cell Physiol.

[18] Zhang Y, Xue W, Li X, Zhang J, Chen S, Zhang JL, Yang L, Chen LL (2016). The biogenesis of nascent circular RNAs. Cell Rep.

[19] Geng Y, Jiang J, Wu C (2018). Function and clinical significance of circRNAs in solid tumors. J Hematol Oncol.

[20] Zhou C, Liu HS, Wang FW, Hu T, Liang ZX, Lan N, He XW, Zheng XB, Wu XJ, Xie D (2020). circCAMSAP1 promotes tumor growth in colorectal cancer via the miR-328-5p/E2F1 axis. Mol Ther.

[21] Sang Y, Chen B, Song X, Li Y, Liang Y, Han D, Zhang N, Zhang H, Liu Y, Chen T (2019). circRNA_0025202 regulates tamoxifen sensitivity and tumor progression via regulating the miR-182-5p/FOXO3a axis in breast cancer. Mol Ther.

[22] Tornesello ML, Faraonio R, Buonaguro L, Annunziata C, Starita N, Cerasuolo A, Pezzuto F, Tornesello AL, Buonaguro FM (2020). The role of microRNAs, long non-coding RNAs, and circular RNAs in cervical cancer. Front Oncol.

[23] Hu C, Wang Y, Li A, Zhang J, Xue F, Zhu L (2019). Overexpressed circ_0067934 acts as an oncogene to facilitate cervical cancer progression via the miR-545/EIF3C axis. J Cell Physiol.

[24] Strazisar M, Cammaerts S, van der Ven K, Forero DA, Lenaerts AS, Nordin A, Almeida-Souza L, Genovese G, Timmerman V, Liekens A (2015). MIR137 variants identified in psychiatric patients affect synaptogenesis and neuronal transmission gene sets. Mol Psychiatry.

[25] Silber J, Lim DA, Petritsch C, Persson AI, Maunakea AK, Yu M, Vandenberg SR, Ginzinger DG, James CD, Costello JF (2008). miR-124 and miR-137 inhibit proliferation of glioblastoma multiforme cells and induce differentiation of brain tumor stem cells. BMC Med.

[26] Chen L, Wang X, Wang H, Li Y, Yan W, Han L, Zhang K, Zhang J, Wang Y, Feng Y (2012). miR-137 is frequently down-regulated in glioblastoma and is a negative regulator of Cox-2. Eur J Cancer.

[27] Chen Q, Chen X, Zhang M, Fan Q, Luo S, Cao X (2011). miR-137 is frequently down-regulated in gastric cancer and is a negative regulator of Cdc42. Dig Dis Sci.

[28] Zhu X, Li Y, Shen H, Li H, Long L, Hui L, Xu W (2013). miR-137 inhibits the proliferation of lung cancer cells by targeting Cdc42 and Cdk6. FEBS Lett.

[29] Zhang H, Yan T, Liu Z, Wang J, Lu Y, Li D, Liang W (2018). MicroRNA-137 is negatively associated with clinical outcome and regulates tumor development through EZH2 in cervical cancer. J Cell Biochem.

[30] Miao H, Wang N, Shi LX, Wang Z, Song WB (2019). Overexpression of mircoRNA-137 inhibits cervical cancer cell invasion, migration and epithelial-mesenchymal transition by suppressing the TGF-beta/smad pathway via binding to GREM1. Cancer Cell Int.

[31] Nakamura Y, Migita T, Hosoda F, Okada N, Gotoh M, Arai Y, Fukushima M, Ohki M, Miyata S, Takeuchi K (2009). Kruppel-like factor 12 plays a significant role in poorly differentiated gastric cancer progression. Int J Cancer.

[32] Mak CS, Yung MM, Hui LM, Leung LL, Liang R, Chen K, Liu SS, Qin Y, Leung TH, Lee KF (2017). MicroRNA-141 enhances anoikis resistance in metastatic progression of ovarian cancer through targeting KLF12/Sp1/survivin axis. Mol Cancer.

[33] Kim SH, Park YY, Cho SN, Margalit O, Wang D, DuBois RN (2016). Kruppel-like factor 12 promotes colorectal cancer growth through early growth response protein 1. PLoS ONE.

[34] Wang J, Pu J, Zhang Y, Yao T, Luo Z, Li W, Xu G, Liu J, Wei W, Deng Y (2019). DANCR contributed to hepatocellular carcinoma malignancy via sponging miR-216a-5p and modulating KLF12. J Cell Physiol.

