# Supplementary material for: CircNEIL3 promotes cervical cancer cell proliferation by adsorbing miR-137 and upregulating KLF12
Source: Cancer Cell Int. 2021 Jan 7;21:34. doi: 10.1186/s12935-020-01736-4 (PMC7792354; doi:10.1186/s12935-020-01736-4)
Supplement: Supplementary file 1 — Additional file 1: Table S1. Primers for qRT-PCR. Table S2. RNA probes for FISH. [file 12935_2020_1736_MOESM1_ESM.docx]

**Additional Table S1. Primers for qRT-PCR**

| **Gene name** | **Forward primer (5’-3’)** | **Reverse primer (5’-3’)** |
| --- | --- | --- |
| circNEIL3 | ACTGGGGAGAAGCAGTGTTT | TCACTCTTGAGCACTGAATCAT |
| hsa_circ_0000583 | GCCAGTCGCTCTCTTTGATG | ATGTTCACCTGCCGCGTG |
| hsa_circ_0060458 | TGCCTCTCTCTTGTCATCGA | CAGATGATGCGGTCCTCCTC |
| hsa_circ_0090531 | TGGGCTAGGGGAAATAAGGC | CACTTTGATGGCCACTTCTGA |
| hsa_circ_0006968 | CCATCTTACAAGGCCTTTTCAG | CAACCCAAACCTGTGAATTTT |
| hsa_circ_0004266 | GCTTCCTCGTTGTTTGGACA | TGAATACTGAGTTTGCTAGAAGAAGT |
| hsa_circ_0068858 | TCAGCCTGTTCTAACTCCGG | GTGTCTGAAAAGTCACCCAC |
| hsa_circ_0045016 | CCAAGATGAAGTGGGAGCAC | CGTTTGCTTCTAACCAACTTGT |
| hsa_circ_0066970 | ATGTCCCCAGCATCTCCAAT | GAAGCAGGTTTGAAGTCATCTCT |
| hsa_circ_0014756 | CATCAAGATTGGCCTGCTGG | GAGTTCCTCAGCTGCAGAGT |
| MITF | CAGTCCGAATCGGGGATCG | TGCTCTTCAGCGGTTGACTTT |
| PDLIM3 | GGTCATCACCAGGATTACACCA | AAAGCCGTCAATAGCCAGGAT |
| KLF12 | CGGCAGTCAGAGTCAAAACAG | CGGCTTCCATATCGGGATAGT |
| PALM2-AKAP2 | CAGTGACGGACGTGTCCAC | GCAGCACATTCTCCCACCC |
| SS18 | CACCTCCACGCTCTCACAAC | ATCTGGCCGTTCATCTGGTTC |

**Additional Table S2. RNA probes for FISH**

| **Gene name** | **probe sequence** |
| --- | --- |
| circNEIL3 | GAGGAAATACTAAGTCACGAGT |
| miR-137 | CTACGCGTATTCTTAAGCAATAA |
